# Prolonged survival in EGFR exon 20 insertion mutant lung adenocarcinoma: case report of sequential osimertinib and furmonertinib with research trend analysis

**DOI:** 10.3389/fmed.2025.1677737

**Published:** 2026-01-09

**Authors:** Yishan Lu, Ruiguo Zhao, Ning Wang, Jingjing Zhao, Nana Huang, Abdullah Al-danakh, Haijing Liu, Ping Gao

**Affiliations:** 1 Department of Dermatology, First Affiliated Hospital of Dalian Medical University, Dalian, China; 2 Department of Oncology, The Second Affiliated Hospital of Liaoning University of Traditional Chinese Medicine, Shenyang, Liaoning, China; 3 Liaoning Provincial Cancer Hospital, Shenyang, Liaoning, China; 4 Department of Urology, Amran University, Amran, Yemen; 5 Department of Integrative Medicine, Xiangya School of Medicine, Central South University, Changsha, Hunan, China; 6 Department of Oncology, The First Affiliated Hospital of Dalian Medical University, Dalian, Liaoning, China

**Keywords:** EGFR exon 20 insertion, non-small cell lung cancer, osimertinib, furmonertinib, bibliometric analysis

## Abstract

**Background:**

Epidermal growth factor receptor (EGFR) exon 20 insertion (ex20ins)-mutant non-small cell lung cancer (NSCLC) is characterized by limited sensitivity to standard-dose EGFR tyrosine kinase inhibitors (EGFR-TKIs) and historically poor clinical outcomes. Although agents such as amivantamab and other targeted therapies have expanded treatment options, access barriers and marked variant-specific heterogeneity remain major challenges. Emerging evidence suggests that dose-escalated third-generation EGFR-TKIs may provide clinical benefit in selected ex20ins subtypes, yet real-world data are scarce.

**Case presentation:**

We describe a 56-year-old never-smoking female with metastatic lung adenocarcinoma harboring an EGFR A767_V769dup exon 20 insertion and co-mutations in *TP53, SETD2, SMAD4*, and *ETV6*, with low PD-L1 expression. In the setting of limited access to amivantamab at diagnosis and preliminary evidence supporting intensified EGFR inhibition in certain “near-loop” ex20ins variants, the patient received off-label high-dose osimertinib 160 mg once daily as first-line therapy. She achieved a durable partial response with manageable Grade 1 skin and nail toxicities and no dose reductions. Following disease progression, and after multidisciplinary discussion and informed consent, she was switched to off-label furmonertinib 240 mg once daily, resulting in additional disease control. Sequential high-dose osimertinib followed by high-dose furmonertinib yielded an overall survival of approximately 37 months.

**Literature and trend overview:**

To contextualize this case, we conducted a targeted narrative review and a descriptive bibliometric overview using CiteSpace (2000–2023) based on Web of Science Core Collection records. This analysis demonstrated a growing global research focus on third-generation EGFR-TKIs and variant-specific treatment strategies for EGFR ex20ins-mutant NSCLC, supporting the rationale underpinning the therapeutic approach adopted in this report.

**Conclusion:**

Sequential high-dose third-generation EGFR-TKIs may offer clinically meaningful benefit in selected EGFR ex20ins cases; however, this strategy remains non-standard and should be regarded as hypothesis-generating, warranting further prospective evaluation.

## 1 Introduction

Over the past two decades, the identification of epidermal growth factor receptor (EGFR) mutations has profoundly transformed the treatment landscape for non-small cell lung cancer (NSCLC). Targeted therapies, particularly EGFR tyrosine kinase inhibitors (TKIs), have become a cornerstone of care in EGFR-mutated NSCLC, leading to substantial gains in survival and quality of life compared with conventional chemotherapy (1–3).

Among EGFR mutations, exon 19 deletions and the exon 21 L858R point mutation are the most common, together accounting for the majority of EGFR alterations and displaying marked sensitivity to first- and later-generation TKIs (1, 2). By contrast, EGFR exon 20 insertion (ex20ins) mutations comprise a smaller subset of NSCLC and historically have been associated with intrinsic resistance to standard-dose EGFR-TKIs (4–7). Structurally, many ex20ins events occur within the αC–β4 loop and adjacent regions of the kinase domain, inducing conformational changes that hinder TKI binding while preserving kinase activity (6).

Clinically, EGFR ex20ins mutations are often enriched in patients with adenocarcinoma histology and never-smoking status and are associated with poorer outcomes compared with tumors harboring classical sensitizing EGFR mutations (1, 7). For many years, platinum-based chemotherapy remained the standard of care and provided only modest benefit in this setting. More recently, the development of ex20ins-directed agents, particularly the oral TKI mobocertinib and the bispecific anti-EGFR/MET antibody amivantamab, has expanded treatment options for this subgroup (4, 8). Nevertheless, reported median progression-free survival (PFS) with these agents remains limited, and access to amivantamab is heterogeneous across health-care systems (7).

Emerging data suggest that certain atypical EGFR ex20ins variants—particularly those located near the loop region or causing relatively subtle structural perturbations—may retain partial sensitivity to third-generation EGFR-TKIs such as osimertinib, especially when administered at escalated doses (5, 7, 9). In parallel, furmonertinib (AST2818), a brain-penetrant third-generation EGFR inhibitor, has demonstrated promising activity in EGFR-mutated NSCLC, including signals of efficacy in ex20ins disease and in intensified dosing strategies (7, 10–13). Collectively, these observations have prompted interest in dose-escalated third-generation EGFR-TKIs as a potential, albeit non-standard, option for selected patients with EGFR ex20ins-mutant NSCLC.

In this context, we report the case of a never-smoking female patient with advanced lung adenocarcinoma harboring the EGFR A767_V769dup exon 20 insertion who achieved prolonged survival (approximately 37 months) with sequential high-dose third-generation EGFR-TKIs: osimertinib 160 mg once daily followed by furmonertinib 240 mg once daily. Our therapeutic strategy was guided by the “near-loop” location of A767_V769dup, early signals supporting intensified third-generation EGFR inhibition in specific ex20ins variants, and constrained availability of approved ex20ins-directed therapies at the time of management. This case is presented as hypothesis-generating rather than implying definitive superiority of this approach. To further contextualize our experience, we additionally conducted a CiteSpace-based bibliometric overview of research trends and therapeutic developments in EGFR ex20ins-mutant NSCLC.

## 2 Case description

### 2.1 Patient history and initial evaluation

On 20 October 2020, a 56-year-old never-smoking woman presented to our oncology outpatient clinic with a one-month history of progressive chest tightness and exertional shortness of breath. She denied prior chronic illnesses or comorbidities, and there was no family history of cancer. On physical examination, breath sounds were diminished over the left lung field. Initial diagnostic work-up, including chest imaging and assessment of pleural fluid, supported a working diagnosis of lung adenocarcinoma with malignant pleural effusion, prompting comprehensive molecular testing.

Chest computed tomography (CT) revealed a significant left-sided pleural effusion and ipsilateral lung collapse (atelectasis). Following therapeutic thoracentesis and lung re-expansion, repeat imaging ruled out compressive masses but demonstrated multiple bilateral pulmonary nodules and miliary foci, suggesting widespread metastatic disease.

A contrast-enhanced chest CT confirmed diffuse bilateral lung nodules and residual left-sided effusion. Whole-body staging with cranial and abdominal CT and bone scintigraphy (ECT) showed no evidence of extrapulmonary metastasis. Ultrasound-guided thoracentesis was performed, and cytological examination of the pleural fluid confirmed malignant cells. On October 22, 2020, the patient was admitted to the Department of Oncology for further evaluation. Contrast-enhanced CT confirmed diffuse bilateral nodules and persistent left pleural effusion. Whole-body imaging (cranial and abdominal CT, bone scintigraphy) excluded extrapulmonary metastases. Ultrasound-guided thoracentesis was performed, and cytological analysis of pleural fluid revealed malignant cells.

**Figure 1:**
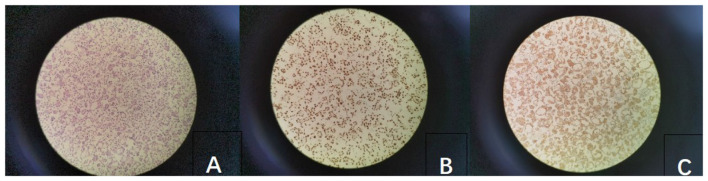
Microscopic morphology: **(A)** Hematoxylin and eosin staining (HE × 200); **(B)** TTF-1 immunohistochemistry (×200); **(C)** Napsin-A immunohistochemistry (×200).

Histopathological evaluation of pleural fluid cell blocks confirmed moderately differentiated adenocarcinoma. Immunohistochemical staining for TTF-1 and Napsin A was strongly positive, supporting the diagnosis of primary pulmonary adenocarcinoma (Figure 1). The disease was staged as IV (TxNxM1a), based on contralateral pulmonary nodules and malignant pleural effusion.

**Figure 2:**
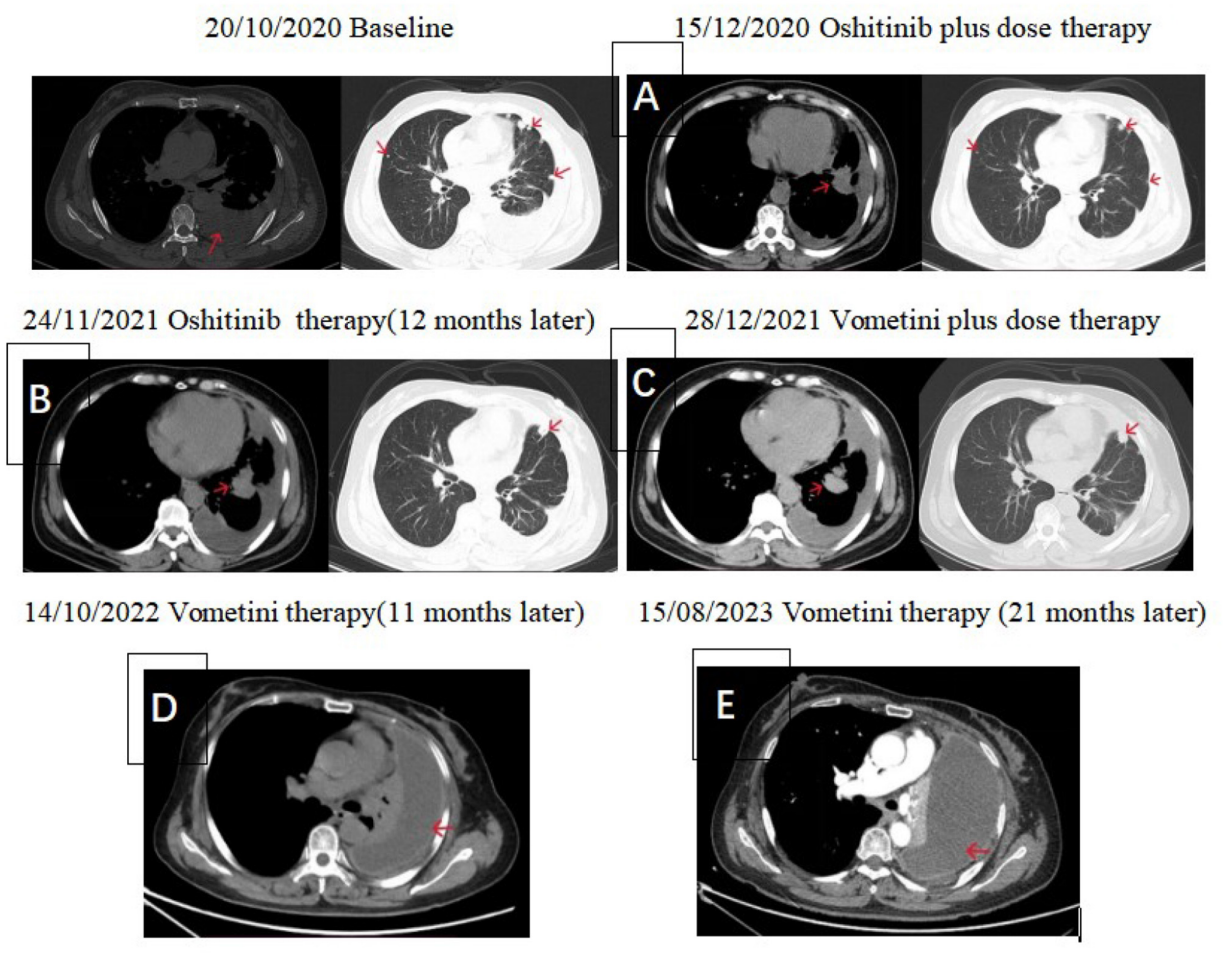
Lung computed tomography images from right to left, respectively, from October 2020 to August 2023. **(A)** After receiving an increased dose of osimertinib, the patient's condition remained stable, the pleural effusion was significantly reduced, the left lung lesions were exposed, and the metastatic lesions in both lungs were reduced. **(B)** After 12 months of increased-dose osimertinib, the primary and metastatic lesions in the left lung were significantly enlarged. **(C)** Following dose escalation of furmonertinib, the lung lesions were largely stable. **(D)** After 11 months, the patient developed a left pleural effusion. **(E)** At 21 months, follow-up revealed recurrence of pleural effusion.

Serial CT imaging from October 2020 to August 2023 documented disease evolution across multiple treatment phases, including high-dose osimertinib and subsequent furmonertinib therapy (Figure 2).

### 2.2 Molecular profiling

Next-generation sequencing (NGS) was performed on pleural effusion and plasma samples using a commercial panel (Nanjing Geneseeq Technology Inc.). A non-frameshift duplication in EGFR exon 20 (c.2300_2308dup, p.A767_V769dup) was identified in the pleural effusion sample with a variant allele frequency (VAF) of 2.2%. Additional somatic alterations included *TP53* (p.K164N, 2.3% VAF), *SETD2* (frameshift, 2.1%), *ETV6* (nonsense, 1.9%), and *SMAD4* (missense, 0.4%). No detectable variants were identified in matched plasma samples. The full NGS profile is summarized in Table 1. NGS results became available in mid-November 2020 and were reviewed by a multidisciplinary team prior to finalizing the targeted treatment strategy.

**Table 1:** Detected somatic alterations and their variant allele frequencies (VAF) in pleural effusion.

**Gene**	**Variant**	**Mutation type**	**Plasma VAF**	**Pleural effusion VAF**
EGFR	A767_V769dup (exon 20)	c.2300_2308dup (non-frameshift)	–	2.2%
ETV6	Q347^*^ (exon 6)	c.1039C>T (nonsense)	–	1.9%
SETD2	S2470Afs^*^17 (exon 19)	c.7404del (frameshift)	–	2.1%
SMAD4	V354E (exon 9)	c.1061T>A (missense)	–	0.4%
TP53	K164N (exon 5)	c.492G>T (missense)	–	2.3%

Plasma testing was negative for all listed mutations. ^*^Indicates the functional class of the genetic variant, such as nonsense, frameshift, or missense mutation.

### 2.3 Immunotherapy biomarker evaluation

Comprehensive profiling of immunotherapy-relevant biomarkers was also conducted. Tumor mutation burden (TMB) was 4.2 mutations/Mb in pleural fluid and 0 mutations/Mb in plasma, consistent with a TMB-low status. Microsatellite instability (MSI) and mismatch repair (MMR) testing revealed no pathogenic alterations. Other genes implicated in response to immune checkpoint inhibitors—including *POLE, POLD1, CDK12, KRAS*, and *PBRM1*—were wild-type. Programmed death-ligand 1 (PD-L1) expression was reported as low/negative (< 1%), further suggesting a limited expected benefit from immune checkpoint inhibitor monotherapy. These results are summarized in Table 2.

**Table 2:** Genomic and immunologic biomarkers related to immunotherapy benefit based on current evidence and FDA approvals.

**Biomarker**	**Test result**	**Immunotherapy relevance**
TMB	Pleural fluid: 4.2 muts/Mb	KEYNOTE-158: TMB-H (>10 muts/Mb) may benefit from immunotherapy.
	Plasma: 0 muts/Mb	
Microsatellite analysis	Not applicable	Patients with microsatellite instability high (MSI-H) can benefit from immunotherapy. Pembrolizumab was approved by the FDA for patients with MSI-H solid tumors. Nivolumab monotherapy/combined with Ipilimumab is used after progression to standard chemotherapy.
MMR genes	No mutations affecting protein function were detected	If MLH1, MSH2, MSH6 and PMS2 mutations cause dMMR, they can be obtained from tumors. Pembrolizumab is FDA approved for dMMR. For patients with body tumors, Nivolumab monotherapy plus Ipilimumab was used for standardization. Patients with dMMR metastatic colorectal cancer after treatment progression can benefit.
PBRM1	No mutations affecting protein function were detected	PBRM1 expression was inversely correlated with cytotoxic gene expression in T cells. PBRM1 loss may be involved in resistance mechanisms.
POLE	No mutations affecting protein function were detected	Mutation was a positive predictor of immunotherapy. POLE mutation may lead to a higher tumor mutation burden, which is positive for immunotherapy.
POLD1	No mutations affecting protein function were detected	POLD1 mutations can lead to higher tumor mutation load, positive for immunotherapy.
TP53	No mutations affecting protein function were detected	TP53 mutation may be a positive predictor of immunotherapy.
KRAS	No mutations affecting protein function were detected	Activating KRAS mutation may be a positive predictor of immunotherapy.
CDK12	No mutations affecting protein function were detected	It was found to be higher in prostate cancer patients with a double copy loss of CDK12. The number of immune cells and neoantigens may be positive predictors of immunotherapy.

### 2.4 Treatment course and response

The patient initially received one cycle of platinum-doublet chemotherapy consisting of pemetrexed (800 mg on day 1) and carboplatin [area under the curve (AUC) 5; 450 mg on day 1] on 2 November 2020. Shortly thereafter, NGS results confirmed the presence of an EGFR exon 20 insertion. Given the historically poor response of this mutation subtype to first- and second-generation EGFR-TKIs, emerging data supporting dose-escalated third-generation TKIs in selected ex20ins variants, and the fact that amivantamab and other ex20ins-directed agents were not yet available or reimbursed in the treating region, a multidisciplinary team recommended high-dose osimertinib (160 mg orally once daily) as off-label targeted therapy. The rationale, off-label nature of the regimen, potential benefits and risks, and alternative options (including continuation of chemotherapy and future antibody-based therapies) were discussed in detail with the patient and her family, and written informed consent was obtained.

Osimertinib was initiated on 23 November 2020. After two months of treatment, imaging demonstrated a partial radiologic response, with a marked reduction in pleural effusion and stabilization of pulmonary lesions. Symptomatically, the patient reported improved dyspnea and performance status. During osimertinib therapy, she developed only Grade 1 paronychia and dry skin, which were managed conservatively without dose interruption or reduction. No Grade ≥2 toxicities were observed. Treatment adherence, assessed through clinic interviews and pharmacy refill records, was estimated to exceed 90%. Overall, the clinical course under osimertinib was characterized by a durable partial response and sustained disease control.

After 12 months of high-dose osimertinib, a follow-up computed tomography (CT) scan on 24 November 2021 revealed an increase in the size of the left lung mass and bilateral pulmonary nodules, indicating disease progression. In light of emerging clinical and pharmacologic data suggesting activity of high-dose furmonertinib in selected EGFR ex20ins variants, and the continued lack of practical access to amivantamab for this patient, therapy was switched to furmonertinib 240 mg orally once daily. This decision was again made in a multidisciplinary setting, and the off-label nature of treatment, potential benefits, and risks were reviewed with the patient, who provided renewed informed consent.

**Figure 3:**
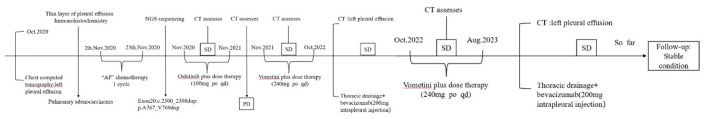
Clinical timeline showing key diagnostic and therapeutic milestones, including initiation of targeted therapy, disease progression, and management of pleural effusion.

Under furmonertinib, the disease remained radiologically stable for an additional 11 months, until October 2022, when imaging revealed recurrent left pleural effusion. The patient underwent thoracentesis and continued furmonertinib at the same dose. She maintained good tolerance, with no new clinically significant adverse events or need for dose modification. In August 2023, pleural effusion recurred and was again drained symptomatically; on this occasion, intrapleural bevacizumab (200 mg) was administered, followed by ongoing furmonertinib therapy. The full timeline of diagnostic evaluations and treatments is summarized in Figure 3.

As of November 2023, the patient remains alive with stable disease, having achieved a progression-free survival (PFS) of 12 months on high-dose osimertinib, followed by durable disease control on high-dose furmonertinib, and a cumulative overall survival (OS) of approximately 37 months from diagnosis. This prolonged survival under sequential high-dose third-generation EGFR-TKI therapy is notable in the context of EGFR exon 20 insertion-mutant NSCLC and underpins the hypothesis-generating nature of this report.

## 3 Discussion

Lung adenocarcinoma is the most common histologic subtype of non-small cell lung cancer (NSCLC), with EGFR mutations detected in approximately 12%–38% of cases, particularly among East Asian populations and never-smokers (3, 14). The two most frequent activating mutations—exon 19 deletions and the exon 21 L858R point mutation—are highly sensitive to approved EGFR tyrosine kinase inhibitors (EGFR-TKIs) and have underpinned the success of targeted therapy in this setting (1, 2). By contrast, EGFR exon 20 insertions (ex20ins) represent a biologically and clinically distinct subset characterized by structural resistance to conventional TKIs and poorer outcomes (6, 15).

Ex20ins mutations introduce additional amino acids into the kinase domain αC-helix or its adjacent loop, perturbing the active-site conformation and impairing inhibitor binding while maintaining or even enhancing EGFR kinase activity (6). Clinically, this translates into shorter progression-free survival (PFS) and overall survival (OS) compared with tumors harboring classical sensitizing EGFR mutations, even when patients receive EGFR-TKIs (1, 2, 7). Historically, platinum-based chemotherapy was the standard of care and conferred only modest benefit in this subgroup.

More recently, ex20ins-directed agents such as mobocertinib and the bispecific anti-EGFR/MET antibody amivantamab have reshaped the therapeutic landscape (4, 8). However, despite regulatory approvals, median PFS with these agents generally remains below 9 months, and primary or acquired resistance is common (4, 8, 16). Furthermore, mobocertinib has been voluntarily withdrawn after a reassessment of its overall benefit-risk profile, leaving amivantamab as the principal approved targeted option for many patients with EGFR ex20ins-mutant NSCLC (14, 17). Access and reimbursement for amivantamab remain heterogeneous worldwide, and not all eligible patients can receive it in a timely manner (14, 16). *Amivantamab has demonstrated clinically meaningful activity in EGFR ex20ins NSCLC–showing an objective response rate of approximately 40% with a median duration of response of about 11 months as monotherapy in CHRYSALIS, and a statistically significant PFS benefit in the first-line PAPILLON trial when combined with platinum-pemetrexed (median PFS 11.4 vs. 6.7 months; HR 0.40) (8, 18). Conversely, for third-generation TKIs, small series and early reports suggest that osimertinib 160 mg can elicit radiologic responses in select “near-loop” ex20ins variants, and high-dose furmonertinib (160-240 mg) has achieved disease control with acceptable tolerability in early-phase/real-world cohorts–supporting a variant- and exposure-dependent signal that remains non-standard (5, 7, 9–13, 15)*. Against this backdrop, our case–with an OS of approximately 37 months under sequential high-dose third-generation EGFR-TKIs–is clinically notable. The co-occurring alterations in *TP53, SETD2, SMAD4*, and *ETV6* highlight the molecular complexity of this case. TP53 co-mutation is frequent in EGFR-mutant NSCLC and has been consistently associated with more aggressive disease biology and inferior outcomes under EGFR-TKIs across multiple cohorts and meta-analyses (19–22). SETD2 loss-of-function perturbs chromatin regulation and DNA repair, promoting genomic instability and shaping the tumor immune microenvironment (23–25). SMAD4 inactivation has been implicated in lung tumorigenesis, epithelial–mesenchymal transition, and metastasis, and correlates with adverse clinical features and poor prognosis in NSCLC (26, 27). ETV6, typically known for fusion events in hematologic malignancies, has also been implicated in oncogenic fusions and transcriptional deregulation in solid tumors (28). Despite these insights, no validated predictive role has been established for TP53, SETD2, SMAD4, or ETV6 alterations specifically in the context of third-generation EGFR-TKIs for EGFR ex20ins disease, and no approved co-targeted therapies exist for these alterations in this setting (15, 16). It is therefore difficult to definitively link the observed durable benefit in our patient to these co-mutations. Instead, they likely reflect underlying genomic complexity and may, if anything, have biased expectations toward worse rather than better outcomes. At a mechanistic level, most EGFR ex20ins mutations reduce TKI binding affinity by stabilizing active conformations and altering the geometric constraints of the ATP-binding pocket (6, 15). Dose escalation of third-generation inhibitors such as osimertinib and furmonertinib may partially overcome this barrier by increasing free drug concentrations at the kinase domain and improving occupancy, particularly for variants that do not induce extreme steric hindrance (5, 7, 29). Recent deep mutational scanning work illustrates how specific EGFR substitutions and insertions can modulate sensitivity not only to third-generation TKIs but also to emerging fourth-generation inhibitors, reinforcing the importance of variant-level interpretation (15, 30). For near-loop variants such as A767_V769dup, available structural modeling and limited functional data support the hypothesis that intensified exposure can restore sufficient inhibitory occupancy—a plausible, albeit still speculative, explanation for the durable disease control observed in our patient.

Taken together, this case does not claim superiority of osimertinib or furmonertinib over approved ex20ins-directed agents such as amivantamab. Rather, it provides hypothesis-generating evidence that: (i) specific ex20ins variants such as A767_V769dup may derive clinically meaningful benefit from intensified third-generation EGFR-TKI exposure; (ii) carefully monitored off-label sequential use of high-dose osimertinib and furmonertinib can be feasible and well tolerated in selected patients; and (iii) variant-level structural and clinical interpretation, alongside real-world constraints on drug access and reimbursement, should be transparently integrated into therapeutic decision-making. Prospective trials and larger molecularly annotated cohorts will be essential to validate whether dose-escalated third-generation EGFR-TKIs can be rationally incorporated into treatment algorithms for EGFR ex20ins-mutant NSCLC.

### 3.1 Bibliometric analysis of research trends

**Figure 4:**
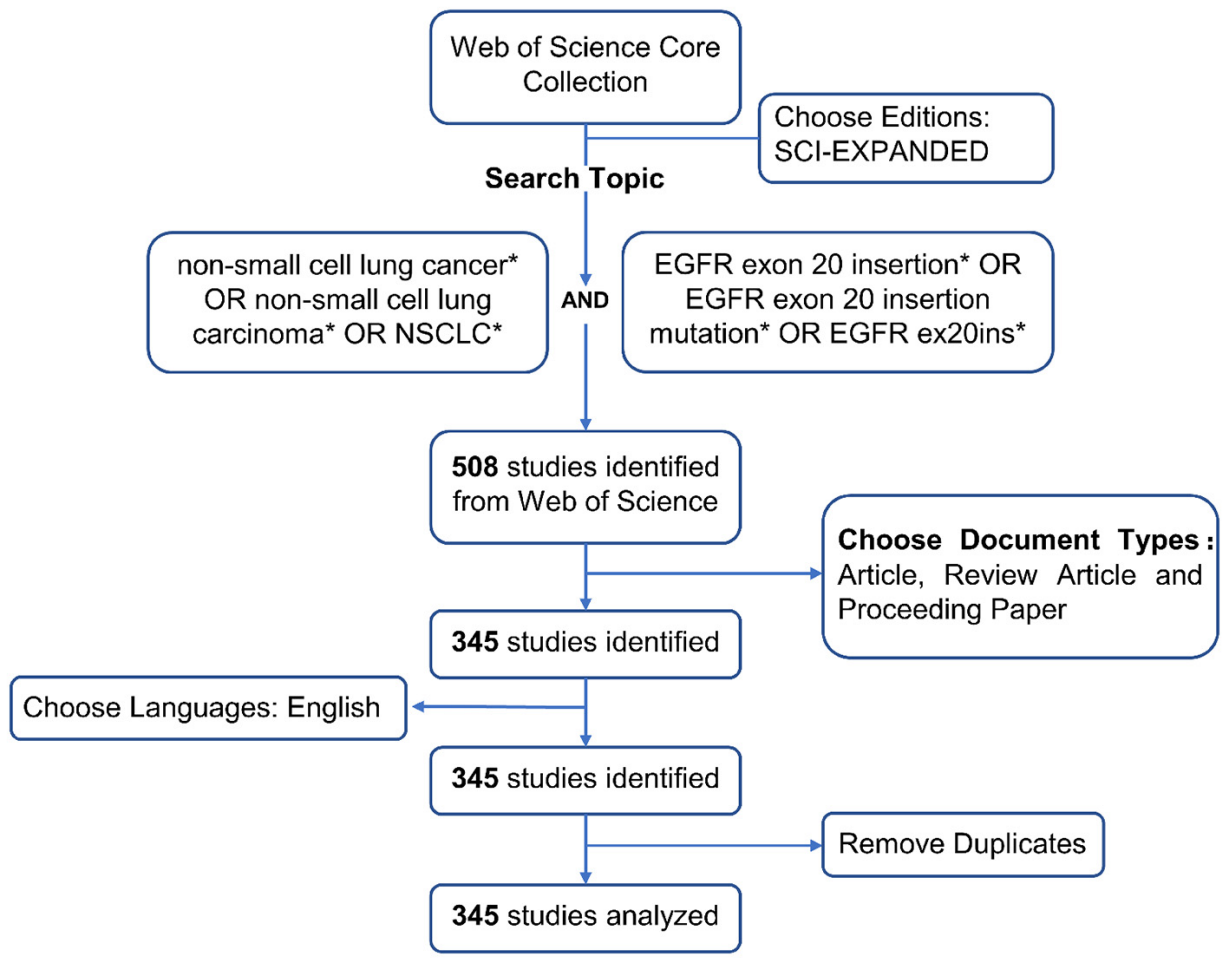
Literature selection workflow for bibliometric analysis of EGFR exon 20 insertion mutations in NSCLC. ^*^Used as a logical operator, meaning “AND / OR”, to show the combination of search terms or criteria used in the literature search strategy.

To contextualize our clinical findings within the broader scientific landscape, we conducted a bibliometric analysis using CiteSpace software to explore global publication trends on EGFR exon 20 insertion (ex20ins) mutations in non-small cell lung cancer (NSCLC) from January 2000 to October 2023. A total of 508 publications were retrieved from the Web of Science Core Collection (WoSCC) using specific search terms related to NSCLC and EGFR exon 20 insertions. The search was restricted to the SCI-Expanded edition, English language, and included only articles, review articles, and proceedings papers. After removing duplicates and non-peer-reviewed documents, 345 eligible studies were included for analysis (Figure 4).

**Figure 5:**
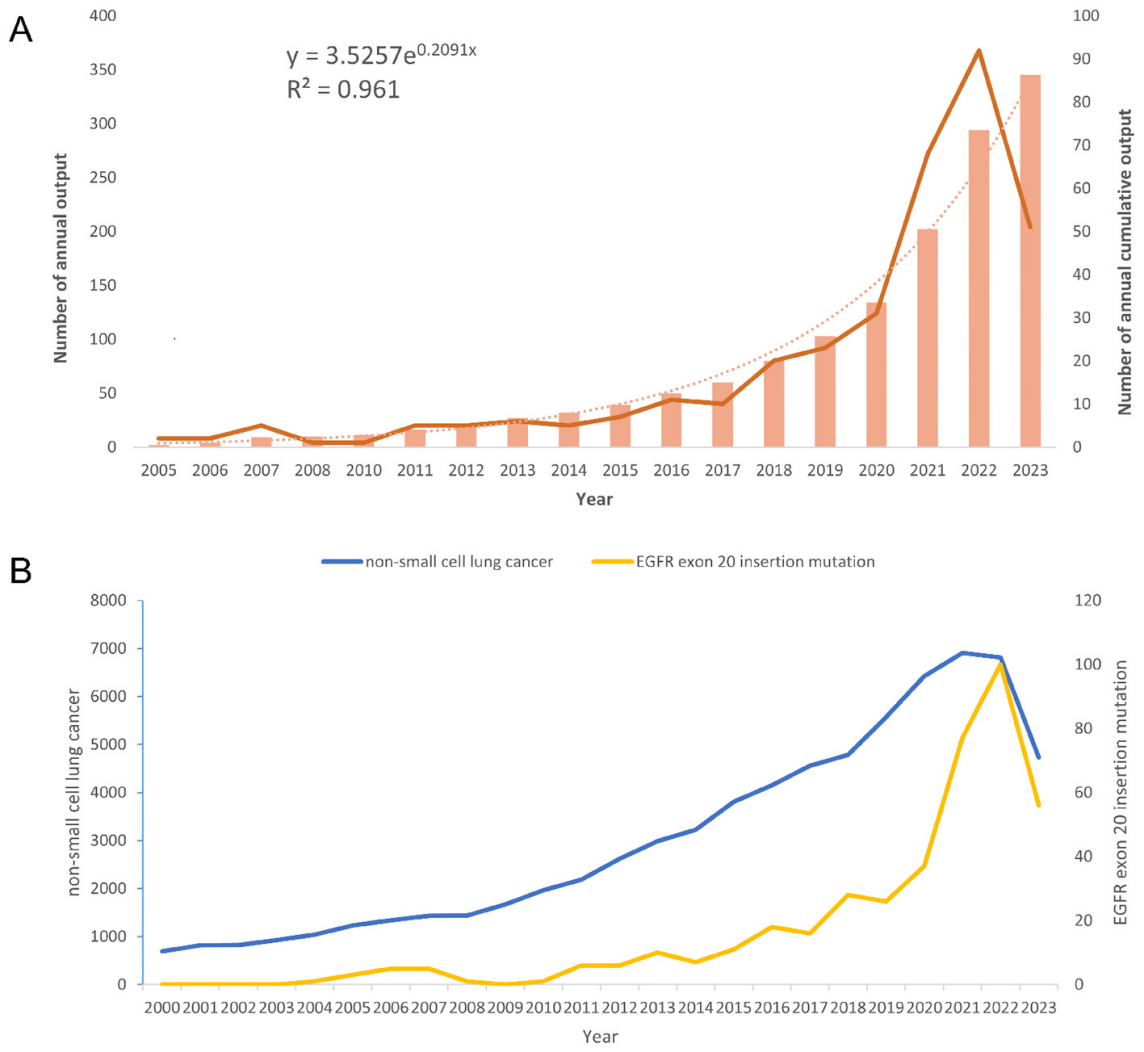
Trends in scientific publications related to EGFR exon 20 insertion mutations in NSCLC. **(A)** Annual and cumulative publication output from 2005 to 2023, with exponential trendline (*R*^2^ = 0.961). **(B)** Comparative publication trends on NSCLC vs. EGFR exon 20 insertion mutations from 2000 to 2023.

The annual number of publications began to rise steadily after 2005 and showed exponential growth, peaking in 2022 (Figure 5A). When comparing the broader topic of NSCLC to the more specific focus on EGFR exon 20 insertions, a substantial surge in ex20ins-related publications was noted after 2018, demonstrating the increasing attention this challenging mutation subset has received in recent years (Figure 5B).

**Figure 6:**
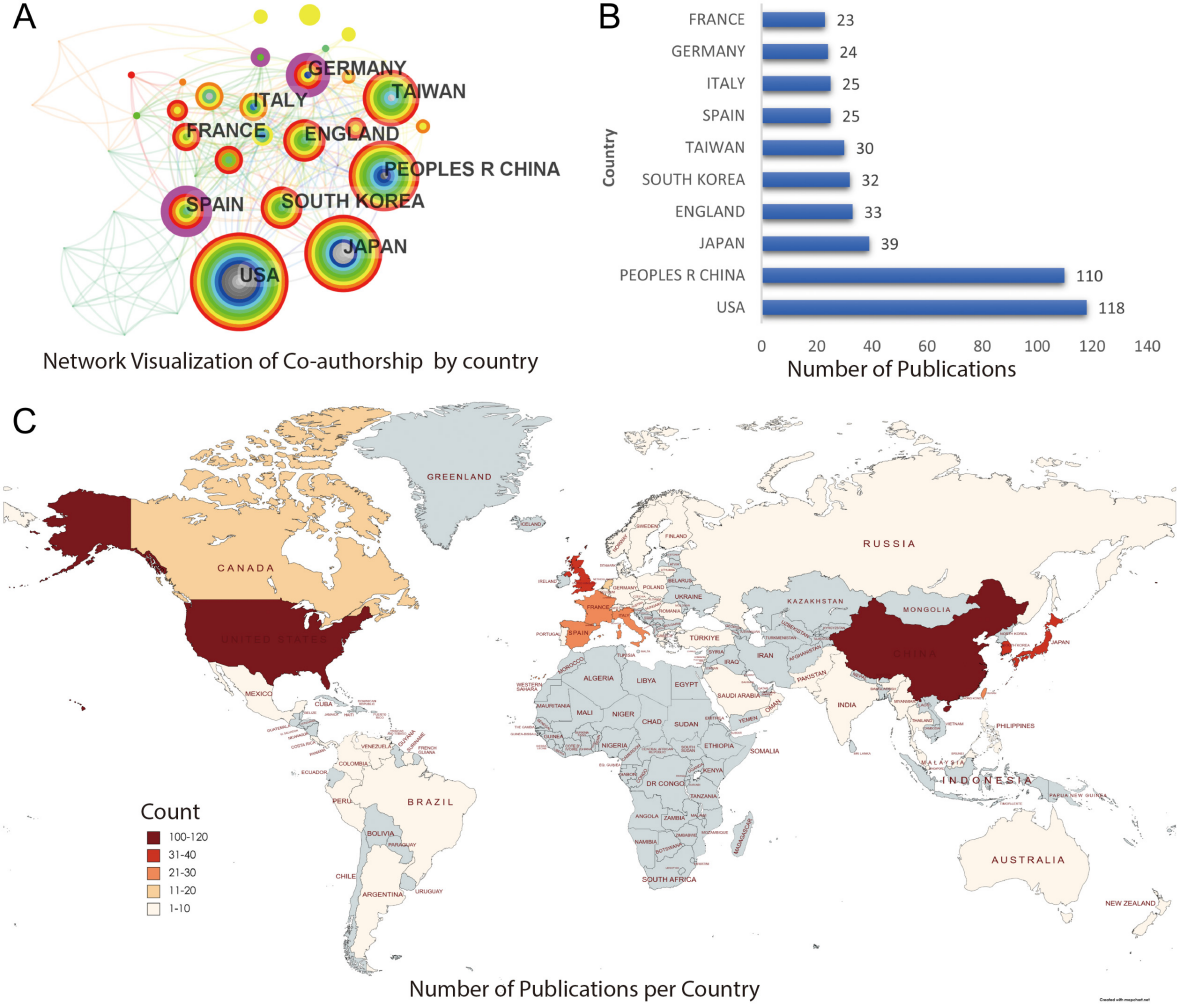
Global geographic distribution of scientific output on EGFR exon 20 insertion mutations in NSCLC. **(A)** Co-authorship network by country. **(B)** Top 10 most productive countries. X-axis represents *Number of Publications (count)*, and the Y-axis lists individual countries. **(C)** World map illustrating publication volume per country, with a color scale labeled “*Publication Count per Country (count)*” to indicate the number of scientific outputs.

Geographic analysis revealed that the United States and China led in publication output, contributing 118 and 110 articles, respectively. Other leading countries included Japan, England, and South Korea (Figure 6). A global co-authorship map highlighted strong research collaboration networks across Asia, Europe, and North America, with central roles occupied by institutions such as Harvard Medical School and Fudan University.

Keyword clustering and citation burst analyses revealed three major research hotspots:

- Evaluation of EGFR-TKIs as a potential replacement for chemotherapy in EGFR ex20ins-mutant NSCLC.
- Structure-based drug design and dose modulation to enhance EGFR-TKI sensitivity.
- Clinical trials assessing third-generation TKIs such as osimertinib and furmonertinib.

**Figure 7:**
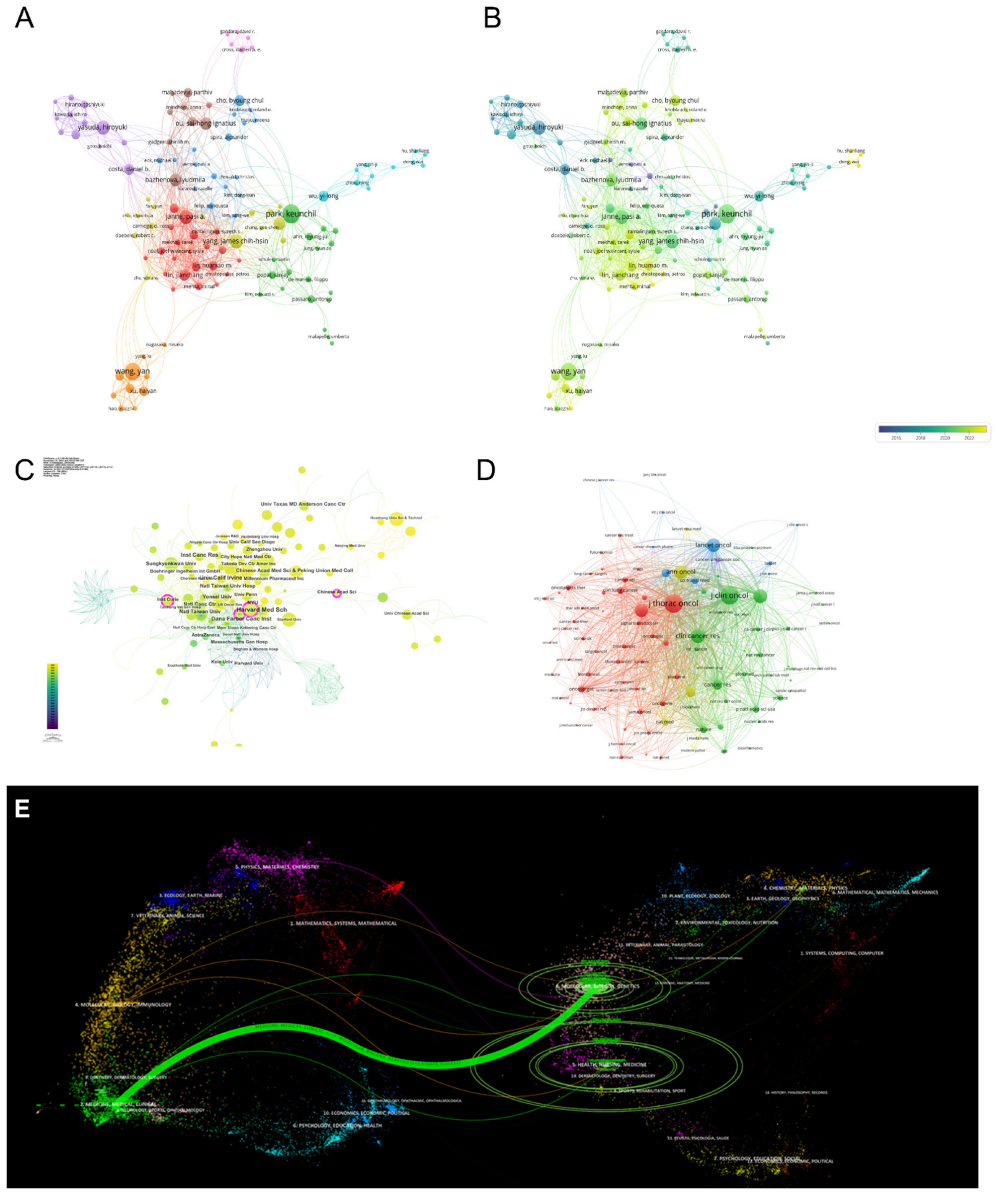
Bibliometric analysis of global research trends on EGFR exon 20 insertion mutations in NSCLC (2000–2023). **(A, B)** Co-citation network of references by theme and year. **(C)** Keyword co-occurrence clusters. **(D)** Institutional collaboration network. **(E)** Timeline and burst detection visualization showing emergent research trends.

The co-citation network (Figures 7A, B), keyword co-occurrence mapping (Figure 7C), and institutional collaboration visualization (Figure 7D) illustrate the evolving research architecture and the central scientific actors. Thematic burst detection further highlighted the rise of precision medicine and dose-adjusted targeted therapy as emerging research frontiers (Figure 7E).

These bibliometric findings are presented as a descriptive supplement rather than a formal systematic review. Their purpose is to contextualize our case within a clearly documented and steadily growing global interest in ex20ins-targeted therapeutic strategies, including third-generation EGFR-TKIs and variant-specific treatment approaches.

### 3.2 Limitations

This case study, although informative, has inherent limitations due to its single-patient design. The prolonged survival observed with sequential high-dose osimertinib followed by high-dose furmonertinib is encouraging but may not be generalizable to all patients with EGFR exon 20 insertions, given the marked molecular and clinical heterogeneity of this subgroup.

The favorable outcome in this patient may relate in part to the specific A767_V769dup variant, which may retain partial sensitivity to third-generation EGFR-TKIs. Co-mutations such as *TP53* and *SETD2*, identified through molecular profiling, were not directly interrogated for prognostic or predictive significance in this context. Furthermore, the absence of molecular reassessment at progression—through tissue rebiopsy or liquid biopsy—limits our ability to characterize resistance mechanisms or clonal evolution under sequential high-dose TKI pressure.

Additionally, the contribution of intrapleural bevacizumab administered late in the treatment course cannot be reliably quantified in the absence of comparative controls. The lack of serial genotyping during progression similarly restricts interpretation of treatment-emergent genomic alterations.

Overall, prospective studies and larger molecularly stratified cohorts are required to validate the clinical utility of sequential high-dose third-generation EGFR-TKIs in EGFR ex20ins-mutant NSCLC and to define optimal therapeutic sequencing algorithms. Until such evidence becomes available, the present report should be regarded as hypothesis-generating and context-dependent, particularly in regions where access to approved ex20ins-targeted therapies remains limited.

## 4 Conclusion

This case highlights the potential role of a sequential high-dose EGFR-TKI strategy in selected patients with advanced NSCLC harboring EGFR exon 20 insertion mutations, particularly the A767_V769dup variant. The patient achieved 12 months of progression-free survival (PFS) on high-dose osimertinib, followed by additional disease control on high-dose furmonertinib, culminating in a total overall survival (OS) of 37 months.

While we do not claim absolute novelty, this represents a comparatively long survival duration for this molecular subgroup and supports the concept that dose-escalated third-generation EGFR-TKIs may provide clinical benefit for selected ex20ins variants when standard therapies such as amivantamab are unavailable or inaccessible. The accompanying bibliometric overview further underscores the growing scientific and clinical interest in novel TKI strategies for this therapeutically challenging population.

Although limited by its single-patient nature, this case adds meaningful real-world evidence and highlights the need for prospective clinical trials evaluating individualized, high-dose therapeutic approaches tailored to specific EGFR exon 20 insertion subtypes and integrated with emerging targeted agents where available.

## Data Availability

The datasets presented in this study can be found in online repositories. The names of the repository/repositories and accession number(s) can be found in the article/supplementary material.
